# Navigating life transitions and mental wellbeing in the digital age: a call for stakeholders to embrace innovation and collaboration

**DOI:** 10.1186/s13034-025-00932-2

**Published:** 2025-06-14

**Authors:** Jörg M. Fegert, Götz Gottschalk, Renee Schneider, Emily Sitarski, Viknesh Sounderajah, Garth Graham

**Affiliations:** 1 Department of Child and Adolescent Psychiatry, Psychosomatics and Psychotherapy, University Hospital of Ulm, Steinhövelstraße 5, 89075 Ulm, Germany; 2 President of ESCAP (The European Society for Child and Adolescent Psychiatry), Kroonlaan 20, Brussel, 1050 Belgium; 3 German Center for Mental Health (DZPG), partner site Ulm, Steinhövelstraße 5, 89075 Ulm, Germany; 4 Competence Center Public Child Mental Health, partner site Ulm, Steinhövelstraße 5, 89075 Ulm, Germany; 5 Google, 1600 Amphitheatre Parkway, Mountain View, CA 94043 USA; 6 YouTube Health, 901 Cherry Avenue, San Bruno, CA 94066 USA

**Keywords:** Life transitions, Mental health, Mental wellbeing, Mental health stigma, Mental health content, Multimedia formats, Digital literacy, Digital media mental health support, Multi-stakeholder approach

## Abstract

Life transitions, such as adolescence, starting higher education or employment, and parenthood, are universally experienced yet psychologically demanding phases that can compromise mental health. While some transitions may promote positive growth, many transitions can also engender significant stress, which can potentially culminate in mental health difficulties and, in some unfortunate instances, psychopathology, especially when individuals lack adequate support. Traditional healthcare systems, already strained by increasing demands and limited resources, frequently offer inflexible, diagnostic-driven pathways with limited accessibility. Therefore, individuals, especially adolescents and emerging adults, have turned increasingly to non-traditional sources for mental health information and support. The internet, in particular, has become an indispensable resource for individuals seeking support around difficult life transitions, including the transition to adulthood. Multimedia content, especially videos, offers unique advantages: cultural sensitivity, peer-shared experiences, the ability to overcome language and literacy barriers and many more which are tailored to individual needs. Innovative content strategies, such as storytelling or expert interviews, can play a pivotal role in reducing the stigma associated with seeking mental health help during life transitions, which may, in turn, increase the likelihood for at-risk individuals to seek formal support if required, and can supplement traditional care with low-threshold information. However, the digital landscape is not without its inherent challenges, including misinformation. While there are frameworks to evaluate and promote credible, evidence-based health content online, there needs to be greater effort to ensure its frictionless integration into formal clinical pathways, when appropriate. To address the complex challenges and fully realize the benefits of digital media mental health care during life transitions, a coordinated, multi-stakeholder approach is essential: this includes collaboration between healthcare providers, policymakers, researchers, technology companies and content creators. We should prioritise developing culturally inclusive, engaging, evidence-based content; promoting digital literacy among users and providers; and expanding access to information, support, and self-management tools specifically designed for individuals navigating life’s transitions. Investment in research and further advocacy for policies is necessary to ensure quality and sustainability of content as well as equitable access for a more resilient and mentally healthy society.

## The role of life transitions on mental wellbeing

Despite the increasing number of divides that exist between communities, whether it is through geographical, cultural, political or socio-demographic differences, we are all connected through a shared experience of being human. For most of us, life’s trajectory is invariably punctuated by a series of transitions, encompassing both anticipated developmental milestones, such as adolescence and parenthood, and unforeseen events, such as job loss or bereavement. While some transitions may promote positive growth, many transitions can also engender significant stress, which can potentially culminate in mental health difficulties and, in some unfortunate instances, psychopathology.

Across the world, health systems, bound by a shared struggle of having to accommodate increasing demand with limited resources, typically present a limited number of established and inflexible routes to access care for those experiencing transition-induced mental health struggles. Long waiting lists, limited diversity in provider demographics, strict diagnostic criteria, and a focus on acute rather than preventative care can deter individuals from seeking help or may even create barriers for those who necessitate professional support. One such tangible example that we have all experienced is the transition towards young adulthood. Typically, this transition is marked by the confluence of multiple life stressors, such as moving away from home, starting higher education or seeking employment. In the face of many concurrent challenges, young adults, who are both metaphorically and literally moving away from their established support systems, may struggle to find sources of information and connection, which they may be looking for the first time on an independent basis. It is therefore unsurprising that many in this cohort, as well as many others across the lifespan (Table 1), experience avoidable psychological harm.

**Table 1:** A table detailing common life transitions, normative developmental challenges vs. life events & their potential associated psychopathological challenges

Life Transition	Normative Transitional Psychological Challenge	Potential Associated Psychopathology
Adolescence to young adulthood	Identity formation: Navigating the transition from dependence to independence. Increased autonomy: Making independent decisions and taking responsibility for oneself. Peer pressure: Conforming to social norms and peer expectations. [6]	Anxiety: Concerns about the future, social acceptance, and performance. Depression: Feelings of sadness, hopelessness, and low self-esteem. Substance use: Experimentation with substances as a coping mechanism or for social acceptance. Self-harm: Deleterious coping mechanism when faced with new stressors [7, 8]
Starting college	Adjusting to a new academic and social environment: Adapting to increased academic demands and navigating unfamiliar social settings. Increased responsibility: Managing academic workload, finances, and personal life. (for an overview see e.g. [9])	Stress: Pressure to succeed academically and socially. Anxiety: Worries about grades, relationships, and future prospects. Depression: Feelings of overwhelm, isolation, and inadequacy. ([9]; for an overview see e.g. [10])
Entering the workforce	Finding meaningful employment: Securing a job that aligns with one’s skills, values, and financial needs. Financial independence: Managing personal finances and providing for oneself. (e.g. [11, 12])	Stress: Job search pressures and competition. Burnout: Exhaustion and cynicism due to job demands. Imposter syndrome: Feelings of fraudulence and self-doubt in professional settings. ([13]; for an overview see e.g. [14])
Parenthood	Adjusting to new roles and responsibilities: Assuming the responsibilities of caring for a child and adjusting to parental roles. Sleep deprivation: Disrupted sleep due to infant care demands. [15]	Postpartum depression: Hormonal changes and psychological stressors can lead to depression in new mothers. Parental anxiety: Concerns about the child’s well-being and ability to provide adequate care. ([16, 17]; for an overview see e.g. [18])
Marriage	Adjusting to intimate cohabitation, negotiating shared decision-making, and managing potential conflicts	Marital discord, anxiety disorders (e.g., generalized anxiety disorder) [19]
Job loss	Financial insecurity: Loss of income and concerns about financial stability. Loss of identity and purpose: Job loss can challenge one’s sense of identity and purpose. (for an overview see e.g. [20])	Depression: Feelings of sadness, hopelessness, and worthlessness. Anxiety: Worries about the future and financial security. Grief: Emotional response to the loss of employment. [21, 22]
Retirement	Adjusting to reduced income and social status: Transitioning from active employment to retirement and managing changes in financial and social status. Loss of purpose: Retirement can lead to a sense of loss of purpose and identity. (for an overview see e.g. [23])	Depression: Feelings of sadness, loneliness, and loss of meaning. Loneliness: Reduced social interaction and support networks. [24, 25]
Bereavement	Grief: Emotional response to the loss of a loved one, characterised by intense sadness, longing, and pain. Loss of attachment: Disruption of the attachment bond with the deceased. [26]	Depression: Persistent sadness, hopelessness, and loss of interest in activities. Anxiety: Worries about the future and coping with the loss. Complicated grief: Prolonged and debilitating grief that interferes with daily functioning. [26, 27]
Divorce	Loss of relationship: Dissolution of a significant romantic relationship, leading to emotional distress and separation. Financial challenges: Division of assets and potential financial instability. Co-parenting: Navigating shared custody and responsibilities for children. [28, 29]	Depression: Feelings of sadness, loss, and betrayal. Anxiety: Worries about the future, financial security, and impact on children. Adjustment disorder: Difficulty coping with the stress and upheaval caused by divorce. [30]
Chronic illness diagnosis	Uncertainty and fear: Uncertainty about the future, disease progression, and treatment options. Physical and emotional challenges: Managing symptoms, pain, and limitations caused by the illness. Identity and body image: Acceptance of changes in physical appearance, limitations, and sense of self. ([31]; for an overview see e.g. [32])	Depression: Feelings of sadness, hopelessness, and loss of interest in activities. Anxiety: Worries about health, treatment, and the future. Grief: Loss of health, function, and previous lifestyle. ([33]; for an overview see e.g. [34, 35])

This once ‘invisible’ transitional issue, that exists at the fault lines of health and social care systems, has been increasingly brought to the fore following the COVID-19 pandemic, which served as a stark ‘natural’ experiment to highlight such issues. It has been well documented that individuals who were coping with transitions were particularly hard hit by the social contact restrictions that the pandemic imposed. Many findings on psychosocial stress and COVID-19 were expected early on [1] and have meanwhile been underscored in many studies [2, 3], demonstrating correlative relationships between psychological distress and social distancing. Moreover, social withdrawal compounded with shame stemming from difficulties in coping with the transition to adulthood, led to an increase in self-stigmatisation [4], which may have, in turn, led to a tendency to avoid seeking help in traditional healthcare institutions, which is often experienced as difficult or hopeless anyway due to the increased demand. The *Lancet Psychiatry* Commission on youth mental health [5] describes a global youth mental health crisis that started to develop long before the COVID-19 pandemic. Global megatrends including the digital transformation influence youth mental health in both directions: as a potential source of harm or decreased life satisfaction or as a new approach for prevention and intervention.

## Online mental health content as a resource for navigating life transitions

As a consequence, there has been a growing reliance upon non-traditional sources of information and support. The internet, in particular, has become an indispensable resource for individuals seeking support around various difficult life transitions, including the transition to adulthood, and the associated normative psychological challenges and potential psychopathology (see Table 1). To exemplify this increasing demand for mental health related content, YouTube has witnessed over 35 billion views on mental health videos globally in 2023 alone [36], which represents a sizable proportion of the overall health content that is consumed on the platform. Moreover, in 2023 the platform has noted a 25% year on year increase in the volume of uploads related to mental health. This shift towards consumers proactively seeking their own health information, e.g. in difficult life transitions, on online platforms presents both unprecedented opportunities and unique challenges for the mental health field. The World Health Organization has published guiding principles on online mental health content for young people [37] e.g. accessibility, evidence-based clarity, inclusivity and diversity, emotional relevance etc. In Europe, a diverse socio-cultural landscape determines how information or help seeking individuals engage with online resources [38].

Online platforms, by virtue of their scale, offer a diverse and expansive array of content, including materials tailored to specific cultural backgrounds, lived experiences, and individual needs. This diversity typically surpasses the resources available in traditional healthcare settings, which may be constrained by standardised protocols and limited cultural sensitivity. In particular, multimedia formats, namely video content, offer a potent tool for disseminating high-quality mental health information and fostering engagement. The ability to present information in a multi-sensory format allows for a more immersive and emotionally resonant experience compared to text-based resources alone. Through this modality, individuals can access a wide range of perspectives, connect with relatable role models, and receive expert guidance in formats that are both accessible and engaging. Moreover, there are clear capabilities for video content to transcend language barriers and to cater to diverse learning styles. Innovative content strategies employed on online platforms, such as storytelling, expert interviews, and animated explainer content, can play a pivotal role in reducing the stigma associated with seeking mental health help during life transitions, which may, in turn, increase the likelihood for at-risk individuals to seek formal support if required. In light of the challenges of pragmatically scaling traditional care models, formally leveraging platforms *that individuals already seek health information from* can serve as a crucial crutch to overstretched health systems. For example, there is the potential for young people with a mental health problem to hear and learn healthy coping skills from others with lived experience, which can complement professional care. For example, people taking psychiatric medications may experience side-effects that are not well known or feel their experiences are not given the attention they may deserve. Videos detailing people’s lived experience may be an important source of validation for people struggling with medication side-effects.

## Conclusion: a call for stakeholders

However, the digital landscape is not without its inherent challenges. The sheer volume and heterogeneity of online content necessitates a thoughtful and measured approach as to how we may uphold the quality, accuracy, and evidence-based nature of information that our communities encounter. To this effect, the National Academy of Medicine [39] and the World Health Organization [40], have set forth consensus derived principles highlighting how credible sources of health information may be identified on media platforms. While these principles guide how platforms, such as YouTube, approach scaling information quality to maximise the impact and utility of this library of content, there needs to be greater effort to ensure its frictionless integration into formal clinical pathways, when appropriate. This integration would enable healthcare professionals to confidently recommend specific platforms and resources to their patients, also in line with the transition phase and the possible psychological challenges they are currently facing, which can signpost to health system specific resources and escalation strategies. Furthermore, as shown in Table 1, life transitions and the associated psychological challenges and potential psychopathology demonstrate that people in many life situations can benefit from low-threshold support through online mental health content.

To achieve this, a multi-stakeholder approach is imperative to address the complex challenges and capitalise upon the opportunities that are presented by the digital age. This necessitates collaboration between healthcare providers, policymakers, researchers, technology companies and content creators. As a collective effort, we should aim to prioritise:

- The creation of evidence-based, culturally relevant, multi-language and engaging mental health content in diverse formats specifically tailored to address the unique needs of individuals experiencing transitions.
- Closer strategic partnerships between academia and online platforms and social media channels, which can significantly amplify the visibility and reach of authoritative mental health content relevant to life transitions. Sponsored content, targeted campaigns, and collaborations with influential figures can help disseminate accurate and helpful information to a wider audience.
- Investment in research to evaluate the effectiveness of online mental health interventions and resources specifically designed for individuals in transition, and identifying best practices for content creation, dissemination, and integration into care.
- Further advocacy for policies and funding mechanisms that support the development and dissemination of high-quality digital mental health resources tailored to life transitions. This includes initiatives to improve digital literacy among both healthcare providers and the public, as well as ensuring equitable access to reliable internet connections for all.

The digital media age offers an unprecedented opportunity to revolutionise mental health care by expanding access to information, support, and self-management tools through diverse and engaging modalities specifically designed for individuals navigating life’s transitions. By embracing a multi-stakeholder approach that prioritises collaboration, innovation, and evidence-based practices, we can collectively address the diverse needs of these individuals and ultimately foster a more resilient and mentally healthy society.

## Data Availability

No datasets were generated or analysed during the current study.
